# Comparison of DMA-80 and ICP-MS Combined with Closed-Vessel Microwave Digestion for the Determination of Mercury in Coal

**DOI:** 10.1155/2020/8867653

**Published:** 2020-12-18

**Authors:** Siyu Zhang, Mingxuan Zhou

**Affiliations:** ^1^State Key Laboratory of Coal Resources and Safe Mining, China University of Mining and Technology, Beijing 100083, China; ^2^College of Geoscience and Survey Engineering, China University of Mining and Technology (Beijing), Beijing 100083, China

## Abstract

As one of the most widely used techniques for concentration determination of trace elements in coal, inductively coupled plasma mass spectrometry (ICP-MS) has also been used in several studies for the determination of mercury concentration in coal. ICP-MS after closed-vessel microwave digestion and a Milestone DMA-80 are employed in this study to determine the mercury concentration in coal. Three NIST  standard references of coal samples were selected as references to verify the accuracy of the test results. The Au rinse solution (200 *μ*g/L, 5% HNO_3_) can diminish mercury memory effects to a blank level within 80 seconds. The results showed that ICP-MS can accurately determine the mercury content in mercury standard solutions, but the mercury concentration in most NIST samples after microwave digestion is lower than the detection level of the ICP-MS. The inaccuracy may be due to volatilization of mercury during solid sample digestion process. By contrast, the determined concentrations in NIST samples by the Milestone DMA-80 are very close to the verified values. Therefore, ICP-MS is not recommended to analyze mercury in coal after digestion even in a closed-vessel digestion system, but the mercury direct analyzer (without digestion) is recommended to analyze mercury in coal.

## 1. Introduction

Mercury is a toxic element that can cause serious environmental and human health problems when it is released into the atmosphere. Approximately, 30% of mercury released into the atmosphere each year is from anthropogenic sources, such as mining, burning of fossil fuels, and intentional use of Mercury [1]. Artisanal, small-scale gold-mining activity and coal combustion in power plants are the largest sectors of the anthropogenic emission [1–3]. Mercury occurs in rocks and soils in the earth's crust [1, 4], accounting for about 10% of mercury emissions from natural geological sources. Previously released mercury accumulated in surface soils and oceans, most of which was rereleased due to human activities, and this rereleased mercury accounts for the remaining 60% [1]. Mercury can remain in the air for a long time and be transported for long distances [3, 4]. Humans and wildlife can be exposed to this mercury, and, if it reaches a certain level, it will cause harm to their health. Consumption of aquatic products containing high levels of methylmercury is another common way of people's route of exposure to mercury [4–6]. High levels of methylmercury in the human body can harm the nervous system and other organ systems [7]. Therefore, the emission of mercury must be taken seriously.

Due to the presence of mercury in coal and the volatility of mercury, coal combustion can cause mercury to be released into the atmosphere. The average concentration of mercury in the world hard coal is 0.1 ppm [8], the average concentration of mercury in the United States coal is 0.17 ppm [9], and the average concentration of mercury in Chinese coals is 0.163 ppm [10]. Many researchers have also investigated the origin of mercury in coal. Finkelman (1980) found that Hg in coal is associated with pyrite, probably of secondary origin [11]. Zhou proposed that the origin types of Hg in coal are mainly terrigenous sedimentary type and epigenetic hydrothermal type [12]. Yudovich and Ketris also suggested that the origin of thermal epigenetic Hg in coal mainly occurs in epigenetic Hg-bearing sulfides in coals, syngenetic pyrite, and hydrothermal solutions [13]. Although the concentration of mercury in coal is modest (generally, less than 0.17 ppm compared to 0.5 ppm in the earth's crust), the tremendous volumes of coal consumption each year (almost 3.73 Gt oil equivalent worldwide with about half from China [14]) result in substantial mercury emissions. In addition, millions of tons of in-ground and near surface coal and coal waste burn each year, thus releasing mercury into the atmosphere [15]. In 2017, thermal power accounted for about 62 percent of China's total power generation [16]. Mercury emissions from China's coal-fired power plants are serious and alarming. Attention should be paid to the concentration and occurrence of mercury in coal, which is closely related to mercury emissions from coal. Accurate quantification of mercury in coal is of great importance in assessing the importance of mercury emissions from coal combustion.

Several analytical methods have been used for determination of mercury in solid samples, including neutron activation analysis (NAA) [17–19], cold-vapor atomic absorption spectrometry (CV-AAS) [20–23], cold vapor atomic fluorescence spectroscopy (CVAFS) [24–29], atomic fluorescence spectroscopy (AFS) [22, 30, 31], and direct mercury analyzer based on concepts of cold vapor atomic absorption spectrometry [32–35].Among these techniques, CV-AAS is the most reliable approach and has been widely used for Hg determination in coal [28, 36, 37]. Yudovich and Ketris have pointed out [13] “the best available method for Hg analysis of solids (coal, ash, and sulfate wastes) is EPA-7473 [38] using an instrument such as the DMA-80 (a direct mercury analyzer, Milestone).”

ICP-MS currently is the most common analytical method for concentration determination of trace elements due to its sensitivity and efficiency [39–44]. Some researchers used ICP-MS or ICP-MS-related technologies to determine Hg concentration in coal and coal fly ash, utilizing techniques such as slurry sampling electrothermal vaporization ICP-MS [45], isotope dilution cold-vapor generation ICP-MS [5], ICP-MS with microwave digestion [46], and ICP-MS after digestion using microwave-induced combustion [47]. However, most of these analytical methods are too complex to be applied to routine testing [41]. The most serious problem when using ICP-MS for mercury analysis that should be addressed is the mercury memory effect [40, 41, 44, 48], because the detection of mercury in solid samples by ICP-MS requires the extraction of mercury from solid samples into aqueous solutions, mercury can adhere to the walls of the sample-introduction system even at very low concentration [39], resulting in a strong memory effort, and therefore, the accuracy of the test results will not be guaranteed.

A number of methods have been developed to eliminate the mercury memory effect during the concentration determination of Hg using ICP-MS, such as gold addition [49, 50], Triton X-100/ammonia/ethylenediaminetetraacetic acid (EDTA) addition [29, 51, 52], hydrobromic acid addition [53, 54], 2-mercaptoethanol (ME) addition in carrier solution [48], and mixing mercury with (NH_4_)_2_H_2_EDTA and the presence of ammonia addition [52].

In this paper, we describe a rinse solution optimized to minimize the mercury memory effect in the shortest possible time, and subsequently, ICP-MS analysis, after closed-vessel microwave digestion, is used to check if this technique can be used to determine mercury concentrations in coal.

## 2. Methods and Materials

### 2.1. Apparatus

#### 2.1.1. ICP-MS and Digestion System

A Thermo Fisher inductively coupled plasma mass spectrometry (X Series II) was used to determine the mercury concentrations in coal samples. The coal samples were digested by using an UltraClave microwave high-pressure reactor (Milestone) prior to ICP-MS analysis. The UltraClave microwave system digests samples by high temperature and high pressure, which can greatly reduce the time required for sample digestion and avoid cross-contamination between samples [55, 56].

#### 2.1.2. DMA-80 Mercury Analyzer

In contrast to the steps of analyzing samples with ICP-MS, the DMA-80 mercury analyzer measures mercury content in the coal sample by direct combustion. The sample was weighed into a metal boat, and the sample contained in the metal tube is dried and thermally decomposed in the oxygen-rich furnace. The generated gases (including mercury and other combustion products, e.g., nitrogen, halogens, and sulfur oxides) are carried to the catalytic furnace and purified by the adsorbent, where various species of Hg are converted into elemental Hg. The mercury is selectively trapped, and the other combustion by-products are flushed off by gold amalgamation. The amalgamation furnace is heated, and mercury is rapidly released. Mercury is carried into the optical cell by oxygen flow for atomic absorption measurement at a wavelength of 253.65 nm. The mercury content in the sample was determined according to Beer's law working curve method. The detection limit of mercury is 0.005 ng, the relative standard deviation (RSD) from eleven runs on a mercury standard reference is 1.5%, and the linearity of the calibration is in the range 0–1000 ng. It will take about five minutes to test one sample by the DMA-80 mercury analyzer.

### 2.2. Reagents, Gases, Standard Solutions, and Investigated Samples

#### 2.2.1. Reagents

MOS (metal-oxide-semiconductor)-reagent HF (40%, v/v), the guaranteed reagent HNO_3_ (65%, v/v), auric standard solution (200 *μ*g/L, 5% HNO_3_), MOS-reagent HCl, the guaranteed reagent H_2_SO_4_ (98%), analytical reagent H_2_O_2_ (30%, v/v), and ultrapure water were used for coal sample digestion. Ultrapure water used during the experiment was prepared by a Milli-Q A10 system (Millipore, 18.2 MΩ·cm). The guaranteed nitric acid and metal-oxide-semiconductor (MOS) reagent hydrofluoric acid were purified by a DuoPUR acid purification system (Milestone) which can reduce the impurities within the acids.

#### 2.2.2. Gases

Ultrapure argon (99.999%) was used as the cooling, auxiliary, and the nebulizer gas. High-purity oxygen used in the DMA-80 mercury analyzer acts both as a carrier gas and as a combustion-supporting gas.

#### 2.2.3. Standard Solutions

Mercury standard solution (1000 *μ*g/ml) was used to prepare five concentration levels (0, 1, 10, 50, and 100 *μ*g/L), which were used to establish calibration curves of mercury. Reagents for microwave digestion and ICP-MS analysis (200 *μ*g/L, 5% HNO_3_) were prepared using an auric standard solution (1000 *μ*g/ml).

The 1000 *μ*g/ml standard ^103^Rh stock solution (GSB 04-1746-2004, National Center of Analysis and Testing for Nonferrous Metals and Electronic Materials) was used to prepare the internal standard (10 *μ*g/L) for online addition during ICP-MS analytical process. The 100 *μ*g/ml standard solution THM-TS-1 (Inorganic Ventures) containing elements lithium, cobalt, indium, and uranium was used to prepare the 1 *μ*g/L tuning solution. All standard solutions were prepared in 2% (v/v) HNO_3_ except for that the rinse solution was prepared in 5% (v/v) HNO_3_.

#### 2.2.4. Investigated Samples

In order to evaluate the suitability and accuracy of the ICP-MS and DMA-80 mercury analyzer for coal mercury concentration determination, three NIST standard references of coal samples, i.e., SRM1632c, SRM2682b, and SRM2685b, were selected as references without further ashing.

### 2.3. Sample Digestion

Samples were digested by the closed TFM vessel UltraClave Microwave High-Pressure Reactor. Each NIST coal sample reference is crushed to <200 mesh (<75 *μ*m) prior to digestion. Four different digestion methods were used to find a way to completely digest the coal samples. Different reagent assemblages are as follows: (1) 50 mg coal sample was added to 5 ml HNO_3_ and 2 ml HF; (2) 50 mg coal sample was added to 5 ml HNO_3_, 2 ml HF, and 2 ml Au standard solution (200 *μ*g/L, 5% HNO_3_); (3) 50 mg coal sample was added to 5 ml HNO_3_, 2 ml HF, and 2 ml Au standard solution (200 *μ*g/L, 5% HNO_3_) was added after microwave digestion; (4) 100 mg coal sample was added to 5 ml HNO_3_, 1 ml HF, and 2 ml HCl. Since hydrofluoric acid was added during digestion, the HF-resistant PFA-made vessels were used during sample digestion and constant volume process.

Nitric acid and hydrofluoric acid are a common reagent combination used for the digestion of coal samples [56–59]. In the previous studies [57, 60], 5 ml of nitric acid and 2 ml of hydrofluoric acid were used to completely digest the coal sample for trace elements concentration determination, and the test results were reliable. Mercury in coal occurs mainly in the following three forms: (1) mercury is related with sulfide (e.g., pyrite); (2) mercury is related with organic matter; (3) mercury is associated with silicate [13, 61–63]. The nitric acid was used to digest organic-associated and sulfide-associated mercury in coal, and the hydrofluoric acid was used to digest silicate-associated mercury in coal sample. In order to prohibit the volatility and adsorption of mercury, 2 ml of gold solution was added to consolidate the mercury in the solution based on the original digestion scheme. In order to prevent mercury from being adsorbed on the wall of the volumetric flask during storage, 2 ml of gold standard solution is added to the solution after microwave digestion. Based on previous studies, the presence of a small amount of hydrochloric acid can dissolve the cinnabar (HgS) in the sample [64]. There are also some studies that use sulfuric acid and hydrogen peroxide in the digestion of samples [55, 65], but, because of the possibility of explosion, these reagents were not used in this study.

The basic load of the digestion tank in the UltraClave reactor consisted of 330 ml ultrapure water, 30 ml H_2_O_2_ (30%, v/v), and 2 ml H_2_SO_4_ (98%, v/v). The initial nitrogen pressure and the maximum temperature were set to 50-bars and 240°C, respectively, for 75 minutes. The microwave-introduced digestion procedure is shown in Table 1. To prevent mercury from volatilizing, the samples were transferred to the PFA volumetric flasks (resistant to HF corrosion) after the digestion was completed and cooled to room temperature. And then the digested samples were diluted with ultrapure water to 100 ml for ICP-MS analysis.

### 2.4. Instrumental Parameters

#### 2.4.1. ICP-MS

The short-term stability test was first performed in standard mode to optimize and test the position of torch and ion lens of the instrument. In order to get a maximum ion signal and stability, a 1 *μ*g/L tuning solution, which was prepared from 10 *μ*g/ml standard solution, was used to tune the facility. The parameters of the instrument after tuning are listed in Table 2. Since hydrofluoric acid causes certain damage to the vitreous sample-introduction system, the spray chamber and the rectangular tube made of hydrofluoric acid-resistant materials were used.

#### 2.4.2. DMA-80 Mercury Analyzer

The critical feature of the Milestone DMA-80 mercury analyzer is that the samples regardless of solid, gaseous, or liquid can be directly measured without any pretreatment. Therefore, the loss of mercury during the sample preparation, mutual contamination, and environmental pollution are avoided, and reliable analytical data are ensured [34, 66]. The entire analytical process of each sample takes only 5 minutes, supplemented by a 40 bit autosampler, and the Windows-based operating software greatly improves the efficiency of the analysis. The parameters of the DMA-80 mercury analyzer are listed in Table 3.

### 2.5. Rinse Solution

One problem with ICP-MS for mercury analysis is the memory effect of mercury during sample aspiration into the ICP-MS facility. In order to diminish the memory effect of mercury, various rinse solutions including ultrapure water, 2% HNO_3_, 2% ammonia solution, 5% HNO_3_, 5% ammonia solution, and Au solution (200 *μ*g/L, 5% HNO_3_) were used to verify the removal mercury memory effect during the aspiration of the liquid sample introduced into the ICP-MS.

When the ICP-MS analyzes trace elements, 2% HNO_3_ is a common rinse solution [40, 67]. We adopted two different concentrations of the HNO_3_ solution, mainly to see the effect of concentration on the removal of memory effects. Different concentrations of ammonium hydroxide were also used to verify the elimination efficiency. The addition of gold solution to the rinse solution is common in the removal of mercury memory effects [52, 53, 68]. This is because Au^2+^ can form gold amalgam with mercury to effectively remove mercury adsorbed on the wall of the injection system [39, 49, 69, 70].

## 3. Results and Discussion

### 3.1. ICP-MS

**Figure 1:**
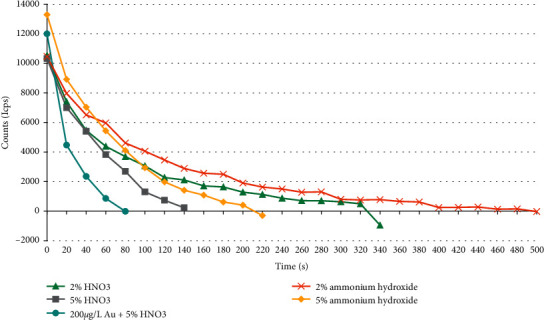
Removal effects of mercury memory by different rinse solutions.

The rinsing effects (Figure 1) by different rinse solutions showed that the 200 *μ*g/L Au^2+^ -5% HNO_3_ [49] rinse solution can effectively eliminate the mercury memory in the shortest time interval of 80 s. By contrast, 2% and 5% HNO_3_ can reach the similar effects at longer time intervals, 320–340 s and 140 s, respectively (Figure 1). As can be seen from Figure 1, the time intervals of 2% and 5% ammonium hydroxide reaching signal blank level are 500 s and 200–220 s, respectively. Therefore, the 200 *μ*g/L Au solution + 5% HNO_3_ was used in this study.

Table 4 shows the determination coefficient of each method and the method detection limit (MDL) of mercury, which is calculated as three times the standard deviation of the average from the blank samples (*n* = 11). As can be seen from Table 4, the linearity of the calibration curve is satisfactory in the range from 0 to 100 *μ*g/L with the determination coefficient *R*^2^ higher than 0.9999 and the method detection limits low. The relative standard deviation (RSD) for each method is obtained by eleven repeated analyzes on NIST standard reference 1632°c (Table 4).

The observed concentrations of the reference materials determined by ICP-MS and the certified values are listed in Table 5, as well as the relative errors between the determined values and the certified values. It can be seen from the test results that the Mercury content of most samples is lower than the detection level, except for SRM2682b and SRM2685b in the second method. However, the measured values differ greatly from the certified values, and the relative errors are high. In order to verify the mercury content in the digested solutions, the digested solutions were measured by the DMA-80 mercury analyzer. The test results obtained by the DMA-80 mercury analyzer are listed in Table 5. From the test results of the DMA-80 mercury analyzer, it can be seen that the content of mercury in the digested sample is lower than the detection level of DMA-80 mercury analyzer or the measured mercury content is close to the blank level.

In this study, ^202^Hg was selected as analytical isotope due to its relatively high content and high sensitivity [71–73]. In the process of analyzing mercury content with ICP-MS, ^103^Rh (10 *μ*g/L) was selected as the internal standard due to its extremely low concentration in coal [57] and its excellent application in the testing of trace elements in coal [57, 60].

During the experiment, 10 ppb and 50 ppb of Hg standard solutions were tested as samples to verify the stability of instrument during the test. The test value of 10 ppb of Hg standard solution was 9.659 (RE = 3.41%), and the test value of 50 ppb of Hg standard solution was 51.7 (RE = 3.40%) and 47.65 (RE = 4.70%). From the test results of the Hg standard solutions, it can be concluded that ICP-MS can accurately test the mercury content in solutions. The rate of internal standard solution recovery of test samples (95.97%–108.14%) indicates that the ^103^Rh (10 *μ*g/L) solution is suitable for use as an internal standard solution.

There are a number of reasons that account for the inaccuracy of the test results: (1) mercury in coal volatilizes during the storage stage [74]; (2) even if the concentration of mercury in coal is very low, it is also easy to adsorb on the wall of container or the surrounding atmosphere [39]; (3) the wall of the containers and the injection system of instrument can be easily penetrated by some mercury species [39, 75]; (4) mercury was adsorbed by colloids or particles in solution [22]; (5) mercury is unstable in polyethylene containers and is easily lost [22, 74–76]. Comparing the test results of ICP-MS and DMA-80 mercury analyzer for the digested solution, the inaccuracy of the test results may be due to volatilization of mercury during the digestion procedure.

### 3.2. DMA-80 Mercury Analyzer

Table 6 shows the results of the samples tested by the DMA-80 mercury analyzer. From the test results, it can be seen that the highest relative error is 15.92%. The relative error of SRM 1632°c is below 1%, and the relative error of SRM 2685b is 5.70%. The DMA-80 mercury analyzer is reliable for the test results for all three samples.

## 4. Conclusions

Although ICP-MS has been used for concentration determination of mercury in coal and could accurately determine mercury in solutions, we do not recommend the use of this technique. Instead, a direct mercury analyzer is the most reliable approach. The major reason for this is mercury's high volatility during sample pretreatment process, even if the digestion is conducted in a closed-vessel digestion system. On the other hand, an optimized rinse solution (Au solution, 200 *μ*g/L, 5% HNO_3_) is recommended to diminish mercury memory in the ICP-MS spray chamber to a blank level within 80 seconds.

## Figures and Tables

**Table 1:** Microwave program for sample digestion.

Step	Time (minute)	T (°C)	P (bar)	Mp (watt)
1	12	60	100	1000
2	20	125	100	1000
3	8	160	130	1000
4	15	240	160	1200
5	60	240	160	1000
Cooling time	60			

*T*, temperature; P, pressure; MP, microwave power

**Table 2:** ICP-MS working conditions.

Item	Value/status	Item	Value/status
Plasma RF power	1400 W	Uptake time	60s
Nebulizer gas flow	1.01 L/min	Washout time	100s
Auxiliary gas flow	0.90 L/min	Pole bias	−6.0 V
Cool gas flow	14 L/min	Hexapole bias	−5.0 V
Sampling depth	100steps	Number of main runs	3
ICP-MS interface	Nickel xt	Dwell time	10 ms
Peristaltic pump speed	30 rpm	Acquisition mode	Peak jumping mode
Spray chamber temperature	2°C	Number of channels	3

**Table 3:** Instrumental parameters of the DMA-80 mercury analyzer.

Item	Value (status)	Item	Value (status)
Injection volume	0.1 g	Carrier gas	Oxygen, 200 ml (min)
Wavelength	253.65 nm	Measuring range	0.0002 ng-30000 ng
Repeatability	RSD<1.5%	Power supply	220 V, 50 Hz
Detector	Silicon-UV photodetector	Detection limit	0.005 ng

**Table 4:** Calibration curves and method detection limit (MDL) of Hg.

Methods	Linearity (ug/L)	Determination coefficient	MDL (ug/L)	RSD (%)
50 mg sample (HNO3 + HF)	0–100	0.999969	1.220	0.34
50 mg sample (HNO3+HF + Au)	0–100	0.999926	1.107	0.29
50 mg sample (HNO3+HF + Au (after))	0–100	0.999929	0.310	0.94
100 mg sample (HNO3+HF + HCl)	0–100	0.999929	1.000	0.10

**Table 5:** Observed and certified values of mercury (*μ*g/kg) in the NIST coal references, as well as internal standard solution recovery (Rec, %) and relative errors (RE, %) of the ICP-MS analysis, and the observed (Obs) values of Hg (*μ*g/kg) in the digested solutions measured by the DMA-80 mercury analyzer.

Methods	Samples	ICP-MS				DMA-80
		Rec (%)	Certified	Observed	^a^RE	Obs
50 mg sample (HNO_3_ + HF)	SRM1632c	106.28	93.8 + 3.7	^b^bdl	/	^b^bdl
	SRM2682b	99.99	108.8 + 2.9	^b^bdl	/	^b^bdl
	SRM2685b	95.97	146.2 + 10.6	^b^bdl	/	^b^bdl

50 mg sample (HNO_3_+HF + au)	SRM1632c	101.48	93.8 + 3.7	^b^bdl	/	^b^bdl
	SRM2682b	104.66	108.8 + 2.9	1651.79	1418.19	^b^bdl
	SRM2685b	104.88	146.2 + 10.6	720.68	79.71	^b^bdl

50 mg sample (HNO_3_+HF + Au (after))	SRM1632c	99.19	93.8 + 3.7	^b^bdl	/	^b^bdl
	SRM2682b	100.36	108.8 + 2.9	^b^bdl	/	0.0064
	SRM2685b	104.51	146.2 + 10.6	^b^bdl	/	0.0089

100 mg sample (HNO_3_+HF + HCl)	SRM1632c	103.26	93.8 + 3.7	^b^bdl	/	0.0082
	SRM2682b	107.61	108.8 + 2.9	^b^bdl	/	0.0192
	SRM2685b	108.14	146.2 + 10.6	^b^bdl	/	0.0192

^a^RE = (|observed-certified|/certified)^∗^100 [60]. ^b^bdl = below detection level.

**Table 6:** Certified (Cer) and observed (Obs) value of mercury in NIST standard reference coal samples.

DMA-80 mercury analyzer	SRM1632c	SRM2682b	SRM2685b
Cer (*μ*g/kg)	93.8 + 3.7	108.8 + 2.9	146.2 + 10.6
Obs (*μ*g/kg)	93.36	91.48	137.87
^a^RE (%)	0.47	15.92	5.70

^a^RE = (|observed-certified|/certified) ^∗^100 [60].

## Data Availability

The data used to support the findings of this study are included within the article.
